# CyberKnife stereotactic radiotherapy for treatment of primary intracranial tumors in dogs

**DOI:** 10.1111/jvim.16086

**Published:** 2021-03-23

**Authors:** Gabrielle L. Carter, Gregory K. Ogilvie, Lenore A. Mohammadian, Phillip J. Bergman, Rachel P. Lee, David R. Proulx

**Affiliations:** ^1^ VCA California Veterinary Specialists Carlsbad California USA; ^2^ Department of Radiation Medicine and Applied Sciences University of California San Diego La Jolla California USA; ^3^ VCA Clinical Studies Los Angeles California USA

**Keywords:** brain, cancer, dog, radiation

## Abstract

**Background:**

Limited data exist about the use, efficacy, and prognostic factors influencing outcome when CyberKnife is used to treat dogs with intracranial neoplasia.

**Objectives:**

To determine the prognosis and associated prognostic factors for dogs that were imaged, determined to have primary intracranial tumors, and treated with CyberKnife radiotherapy.

**Animals:**

Fifty‐nine dogs treated with CyberKnife radiotherapy for primary intracranial tumors.

**Methods:**

Retrospective medical record review of cases from January 2010 to June 2016. Data extracted from medical records included signalment, weight, seizure history, tumor location, tumor type (based on imaging), gross tumor volume, planned tumor volume, treatment dates, radiation dose, recurrence, date of death, and cause of death.

**Results:**

The median progression‐free interval (PFI) was 347 days (range 47 to 1529 days), and the median survival time (MST) was 738 days (range 4 to 2079 days). Tumor location was significantly associated with PFI when comparing cerebrum (median PFI 357 days; range 47‐1529 days) versus cerebellum (median PFI 97 days; range 97‐168 days) versus brainstem (median PFI 266 days; range 30‐1484 days), P = .03. Additionally, the presumed tumor type was significantly associated with MST (P < .001).

**Conclusions and Clinical Importance:**

Use of Cyberknife and SRT might improve MST, compared with RT, in dogs with intracranial neoplasia.

**Abbreviations**
BED: biologically effective dose
cGy: centigray
Cox PH: Cox proportional hazard
CT: computed tomography
GTV: gross tumor volume
Gy: gray
KM: Kaplan‐Meier
MRI: magnetic resonance image
MST: median survival time
PFI: progression‐free interval
PTV: planned tumor volume
SRT: stereotactic radiotherapy

## 1 INTRODUCTION

Diagnosis of intracranial neoplasia in dogs has become more frequent as access to advanced imaging has improved, necessitating a more thorough understanding of treatment options and prognostic indicators. The average age of dogs at diagnosis is 9 years, and golden retrievers and boxer breeds are overrepresented when compared to other breeds.^1^, ^2^ Multiple tumor types fall under the umbrella term of intracranial neoplasia, including meningiomas, gliomas, pituitary tumors, choroid plexus tumors, peripheral nerve sheath tumors, histiocytic sarcoma, hemangiosarcoma, carcinoma, and lymphoma.^3^ Many are solitary primary tumors, but metastatic disease is possible.^1^

Treatments for primary intracranial tumors are aimed at controlling clinical signs and reducing tumor size to minimize compression of surrounding healthy tissues. Treatment options include medical management, surgery, radiation therapy, and a combination thereof.^1^ Median survival time (MST) for dogs treated with medical management alone is 70 days.^4^ While intracranial surgery is an option, poor accessibility and consequent complications often limit its feasibility. Median survival time for dogs undergoing surgical removal of intracranial meningiomas is 120 to 210 days.^4^, ^5^ Adjuvant radiation, after surgical excision of an intracranial meningioma, improves outcomes (MST of 16.5 months).^6^ Radiation therapy offers a less invasive treatment option with a similar MST to surgery. Median survival time for dogs treated with radiation therapy is 5 to 23 months,^4^, ^5^, ^7^ and the use of stereotactic radiotherapy (SRT) for dogs with meningiomas yielded an MST of 561 days.^4^ Additionally, stereotactic radiation surgery for primary intracranial tumors has resulted in MST of 399 to 519 days.^7^, ^8^

Traditional RT protocols for intracranial tumors involve 45 to 54 Gray (Gy) delivered over 15 to 20 fractions.^9^, ^10^ SRT treats tumors with a high degree of accuracy in fewer fractions, necessitating fewer anesthetic events and therefore lower anesthesia associated risk. CyberKnife is 1 type of SRT system, employing real‐time image guidance and a synchronized tumor tracking system to deliver radiation dynamically with submillimeter accuracy.^11^, ^12^ By sparing healthy tissues, fewer early and late adverse radiation effects are anticipated.^11^, ^13^

The purpose of our study is to determine the prognosis and any prognostic factors for dogs with primary intracranial tumors treated with CyberKnife radiation therapy. We hypothesize that dogs treated with CyberKnife have an MST equal to or greater than published MST for dogs treated with other forms of radiation therapy due to the increased accuracy feasible with this system. Such accuracy allows for the delivery of a higher effective dose to the tumor while reducing the dose received by critical late‐responding tissues.

## 2 MATERIALS AND METHODS

Medical records were retrospectively reviewed for all dogs receiving CyberKnife radiation for the treatment of primary intracranial tumors from January 2010 to June 2016. Fifty‐nine dogs were included. Complete data for presenting clinical signs were not available in all cases. Seizure activity was reported, before treatment, for 36 of the dogs.

### 2.1 Pretreatment evaluation

Each dog underwent a physical exam and staging of disease before treatment. Most dogs were staged using complete blood count, biochemistry panel, thoracic radiographs, and abdominal ultrasound. Some dogs were staged using thoracic and abdominal computed tomography (CT) rather than radiographs and ultrasound. Presumptive diagnosed were made via advanced imaging (either magnetic resonance image [MRI] or CT) by a board‐certified veterinary radiologist based on accepted imaging criteria for intracranial neoplasms.^14^, ^15^, ^16^ Each dog had a CT examination of the head before treatment for radiation planning purposes.

### 2.2 Treatment planning, regimen, and monitoring

Noncontrast CT images were used as primary CT set for dose calculation. Computed tomography slice thickness was 1.25 mm to allow accurate distinction between tumor and normal tissues. Contrast CT and MRI images were aligned with the noncontrast primary CT set. Fifty‐four of the 59 dogs had both CT and MRI. The remaining 5 dogs had CT only. Clinical target volume was defined as the gross tumor volume (GTV) delineated on the contrast CT and/or MRI images plus 2 to 2 and a half millimeters. This variation was based on clinician experience considering tumor margins, presumed tumor type, location, and peri‐tumoral structures for each dog. No additional expansion was made for planning tumor volume (PTV). Critical structures, including eyes, optic nerves, optic chiasm, and normal brain, were also contoured. Inverse planning was performed using the Accuray CyberKnife treatment planning system. The dosing goal was 100% of the radiation prescription to 95% of the PTV when achievable. Eyes were normally blocked from beams and the dose constraints to the optic nerves, optic chiasm, healthy brain tissue, and tuning structures were established based on recommendations by the American Association of Physicists in Medicine Task Group 101 Report for human dosing. Tuning structures, which reduce uncontrolled dose diffusion,^17^ were generated during planning to allow for increased conformity to PTV and avoidance of critical structures. Maximum dose to the optic chiasm was limited to less than 0.1 cc receiving greater than 2300 centigray (cGy). Maximum dose to the optic chiasm was limited to less than 500 cGy. In most cases, the best conformality was generally achieved using a single collimator with an isocentric technique although variable collimator iris dimensions were often used. The CyberKnife machine used in our study is equipped with an iris variable aperture collimator (apertures range from 5 to 60 mm) which allows the treatment field to vary within a single treatment. All radiation plans were reviewed and approved by an American College of Veterinary Radiology (ACVR) board‐certified veterinary radiation oncologist.

Treatments were administered via 3 equal doses of 700 to 850 cGy per dose. The total dose per dog ranged from 2100 to 2550 cGy (1 dog received 2100 cGy, 3 dogs received 2250 cGy, 52 dogs received 2400 cGy, and 3 dogs received 2550 cGy). All dogs treated after March of 2012 received 3 doses of 800 cGy, totaling 2400 cGy.

Anesthetic protocols were determined based on a dog's overall health. However, dogs were generally induced with propofol (5.5 mg/kg IV to effect) and maintained on isoflurane/O_2_. All dogs received mannitol (0.5‐1 g/kg IV) before or during treatment. All dogs were mechanically ventilated with an electronic table‐top ventilator (Engler). Anesthetic monitoring during treatment was conducted via live video monitoring. Anesthetic variables (heart rate, breaths per minute, end‐tidal CO_2_, and blood pressure) were monitored via a Cardell 9500HD veterinary monitor and a separate pulse oximetry unit.

### 2.3 Assessment of response to treatment

Repeat physical examinations were performed 2‐ and 4‐weeks after treatment. In some cases, distance from the primary hospital precluded an in‐hospital re‐examination. In these cases, repeat physical examinations were completed by the referring veterinarian. Additional diagnostics and examinations were performed based on the dog's clinical status. These diagnostics did not always include advanced imaging (MRI or CT). However, in 22 cases, repeat advanced imaging was performed after treatment. Advanced imaging was done at least 30 days after CyberKnife treatment (median 247.5 days; range, 30‐1544 days).

### 2.4 Assessment of clinical status and treatment satisfaction

Owners were instructed to continue to follow‐up with either our oncology service or their referring veterinarian after having the 4‐week reexamination. Reexamination intervals were determined by individual clinicians. Owners were advised to monitor dogs for seizures, abnormal mentation, lethargy, and anorexia.

### 2.5 Statistical analysis

Median progression‐free interval (PFI) and ST were determined via Kaplan‐Meier (KM) analysis with Statview statistical analysis software (version 14.3). Progression‐free interval was defined as the number of days from the last treatment to recurrence of clinical signs. ST was defined as the number of days from the last treatment to death due to any cause. The KM method was also used to evaluate categorical/nominal variables significantly influencing PFI and ST. Cox proportional hazards (Cox PHs), univariate analysis, and multivariate stepwise analysis (with all *P* < .2 univariate factors included in the model) were used to evaluate categorical/nominal and numerical/continuous variables significantly influencing PFI and survival. Categorical/nominal variables evaluated include age (≤9.5 years vs >9.5 years), sex, neuter status, breed, weight (≤47 pounds vs >47 pounds), history of seizures, tumor location (cerebrum vs cerebellum vs brainstem and intra‐axial vs extra‐axial), presumed tumor type, consecutive days of treatment, total radiation dose (≤2400 cGy vs >2400 cGy), recurrence, and tumor size on follow‐up imaging (no change vs smaller vs larger). Numerical/continuous variables evaluated include age, weight, GTV, PTV, dose per treatment, total dose, PFI, days from recurrence to date of death, and MST. Dogs were censored from PFI analysis if they had no recurrence of clinical signs, no evidence of progression on imaging, or were lost to follow‐up. Dogs were censored from survival time analysis if they were alive, death was due to another cause, or were lost to follow‐up.

## 3 RESULTS

### 3.1 Dog sample

Fifty‐nine dogs were treated from January 2010 to June 2016. All dogs were examined by 1 board‐certified radiation oncologist. All radiation planning was performed by a radiation physicist in conjunction with 1 veterinary radiation oncologist. The average age of these dogs was 9.5 years old. The most common breeds treated, in order of frequency, included: mixed breed dogs (n = 15), golden retrievers (n = 8), Boston terrier (n = 4), boxer (n = 4), French bulldog (n = 4), English bulldog (n = 3), pug (n = 3), and Shih Tzu (n = 3). Remaining breeds were represented by only 1 dog per breed. Fifty‐six percent (n = 33) of dogs were male, with 94% (n = 31) of those being neutered. Of the female dogs in our study sample, 96% (n = 25) were spayed.

One dog had a biopsy confirmed meningioma. All other tumors were presumptively diagnosed via advanced imaging (CT or MRI) by a board‐certified veterinary radiologist. Meningioma was the most common presumptive diagnosis, with 33 dogs (56%) having this common neoplasm. Other diagnoses include 16 dogs (27%) diagnosed with a presumptive glioma, 7 dogs (12%) diagnosed with a presumptive pituitary macroadenoma, 2 dogs (3%) diagnosed with a presumptive choroid plexus tumor, and 1 dog (2%) diagnosed with a presumptive basilar meningioma or pituitary tumor. The location of tumors was also determined via advanced imaging. Tumors were classified as extra‐axial (71%, n = 42) or intra‐axial (29%, n = 17), and further subdivided based on location within the cerebrum, cerebellum, or brainstem. Thirty‐three dogs (56%) had tumors of the cerebrum, while 4 (7%) had tumors in the cerebellum, and 22 (37%) had tumors in the brainstem.

Because of the retrospective nature of this study, there were variations in treatment protocols before dogs undergoing CyberKnife. These were generally concerning the administration of antiepileptics and corticosteroids. One dog with a presumed meningioma received a dose of palliative radiation (4 Gy) before CyberKnife treatment. Concurrent or subsequent disease seen in this sample included: 1 dog with a thyroid mass at the time of treatment, 1 dog with a splenic stromal sarcoma (treated with splenectomy and carboplatin) after treatment, 1 dog with a histiocytoma of the body wall after treatment, 1 dog with a heart‐base mass (treated with palliative radiation therapy) after treatment, and 1 dog with a hepatocellular carcinoma (surgically removed) after treatment. Additionally, 1 dog was euthanized due to the owner's death with no reported health concerns for the dog.

### 3.2 Treatment course and response

All dogs received 3 fractions of radiation. Generally, treatments were given over 3 consecutive days. Eleven dogs received fractions on nonconsecutive days due to scheduling. One of these delays was due to hospital closure over a holiday. The remaining delays were due to owner preference. Of these dogs, the longest delay involved 2 dogs which received 3 treatments over 8 days.

**FIGURE 1:**
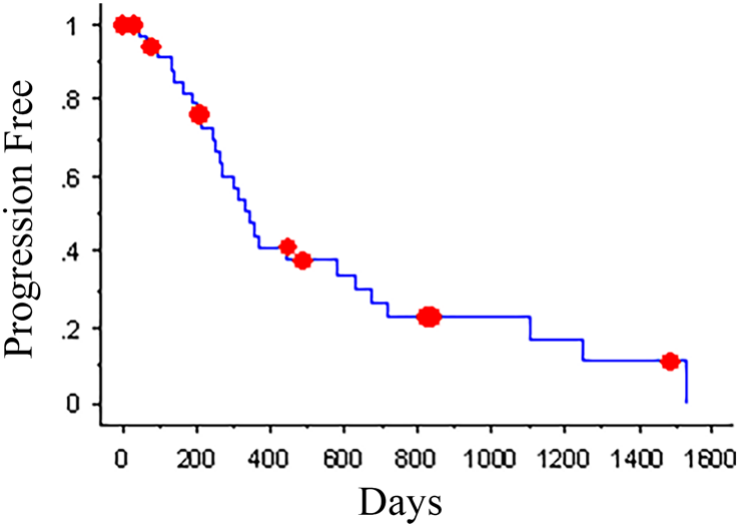
Kaplan‐Meier progression‐free interval plot for dogs with intracranial neoplasia treated with CyberKnife radiation therapy. Kaplan‐Meier overall median progression‐free interval was 347 days. The dots represent censored dogs

Medical treatments after radiation were based on individual clinical signs and included no treatment, corticosteroid administration, antiepileptics, and antiemetics. Recurrence of clinical signs was reported in 27 dogs (range, 47‐1529 days after treatment). Clinical signs included seizure activity, ataxia, circling, head tilt, facial paralysis, strabismus, nystagmus, blindness, and behavior changes. Of these dogs, 3 were still alive at the time of study closure (133‐789 days after the recurrence of clinical signs), and 2 were lost to follow‐up (147 and 181 days after the recurrence of clinical signs). The median PFI was 347 days (Figure 1). Twenty‐two dogs had repeat imaging; this included 12 presumed meningiomas, 6 presumed gliomas, 2 presumed pituitary macroadenomas, and 2 presumed choroid plexus tumors. Imagining was performed due to recurrence of clinical signs in 13 cases and considered routine follow‐up in the remaining 9 dogs. Imaging studies were compared using RECIST criteria.^18^ One study was excluded as initial images were not available for review. Imaging findings included 1 complete response (mass was resolved on repeat MRI performed 218 days after treatment), 11 partial responses, 6 stable disease, and 3 progressive disease.

### 3.3 Prognostic indicators

Mixed breed dogs (n = 15) and golden retrievers (n = 8) were the most common breeds in our sample. Golden retrievers represented 13% of dogs in our study; of these, 7 were diagnosed with a suspect meningioma, and 1 was diagnosed with a suspect glioma. To determine the prognostic influence of age, dogs were divided into 2 groups: those over 9.5 years of age and those less than or equal to 9.5 years of age when starting treatment. Neither breed nor age significantly impacted PFI or ST. Additional prognostic indicators evaluated but not found to significantly impact PFI or ST include sex, neuter status, weight, seizure history, GTV, PTV, consecutive treatment days, and the dose of radiation (Tables 1 and 2).

**TABLE 1:** List of prognostic indicators evaluated for association with disease free interval —univariate analysis

Variable	*P* value
Age	.2
Age (≤9.5 y/>9.5 y)	.17
Sex (M/F)	.46
Neutered (Y/N)	.96
Weight (≤47 lbs, >47 lbs)	.56
Seizure history (Y/N)	.74
Tumor location: brainstem	.95
Tumor location: cerebellum	.009
Tumor location: intra‐axial/extra‐axial	.73
Presumed tumor type: choroid plexus tumor	.35
Presumed tumor type: glioma	.49
Presumed tumor type: macroadenoma	.97
Presumed tumor type: meningioma	.57
Presumed meningioma (Y/N)	.86
Mean cGy to GTV	.34
Mean cGy to PTV	.28
Volume of GTV	.49
Volume of PTV	.61
Consecutive treatment days (Y/N)	.8
Dose per treatment (cGy)	.71
Total dose (cGy)	.71
Total dose (≤2400 cGy/>2400 cGy)	.67
Follow‐up imaging (Y/N)	.23
Follow‐up imaging results	.73

Abbreviations: cGy, centigray; GTV, gross tumor volume; PTV, planned tumor volume.

**TABLE 2:** List of prognostic indicators evaluated for association with ST—univariate analysis

Variable	*P* value
Age	.31
Age (≤9.5 y/>9.5 y)	.25
Sex (M/F)	.6
Neutered (Y/N)	.56
Weight (≤47 lbs, >47 lbs)	.51
Seizure history (Y/N)	.35
Tumor location: brainstem	.12
Tumor location: cerebellum	.89
Tumor location: intra‐axial/extra‐axial	.003
Presumed meningioma (Y/N)	.001
Mean cGy to GTV	.03
Mean cGy to PTV	.2
Volume of GTV	.97
Volume of PTV	.7
Consecutive treatment days (Y/N)	.7
Dose per treatment (cGy)	.78
Total dose (cGy)	.78
Total dose (≤2400 cGy/>2400 cGy)	.36
Follow‐up imaging (Y/N)	.16
Follow‐up imaging results	.89

Abbreviations: cGy, centigray; GTV, gross tumor volume; PTV, planned tumor volume.

**FIGURE 2:**
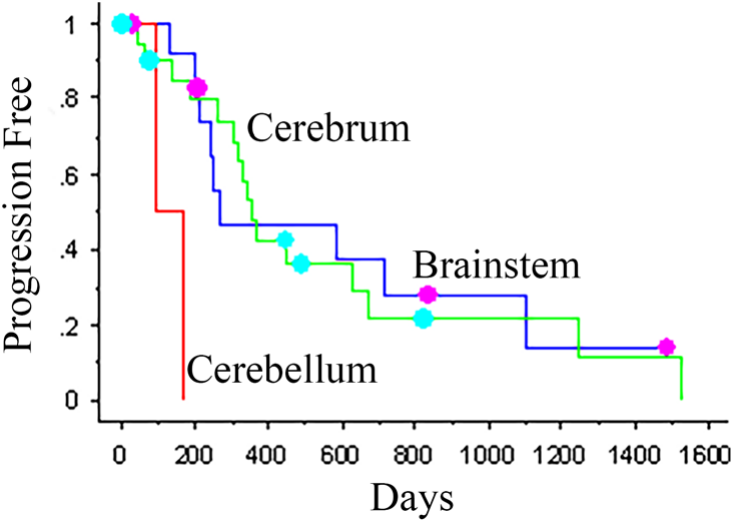
Kaplan‐Meier progression‐free interval plot for dogs with cerebral, cerebellar, and brainstem neoplasia treated with CyberKnife radiation therapy. The dots represent censored dogs

The location of the tumor was significantly associated with PFI when comparing cerebrum vs cerebellum vs brainstem (Cox PH multivariate analysis, *P* = .03). Dogs with tumors in the cerebrum had a median PFI of 357 days, and dogs with brainstem tumors had a median PFI of 266 days, while dogs with cerebellar tumors had a median PFI of only 97 days (*P* = .01) (Figure 2). Using the Cox PH multivariate analysis, the cerebellar hazard is 9.824 (95% confidence interval); thus, dogs with cerebellar tumors are over 9 times more likely to experience shorter PFIs than other dogs.

**FIGURE 3:**
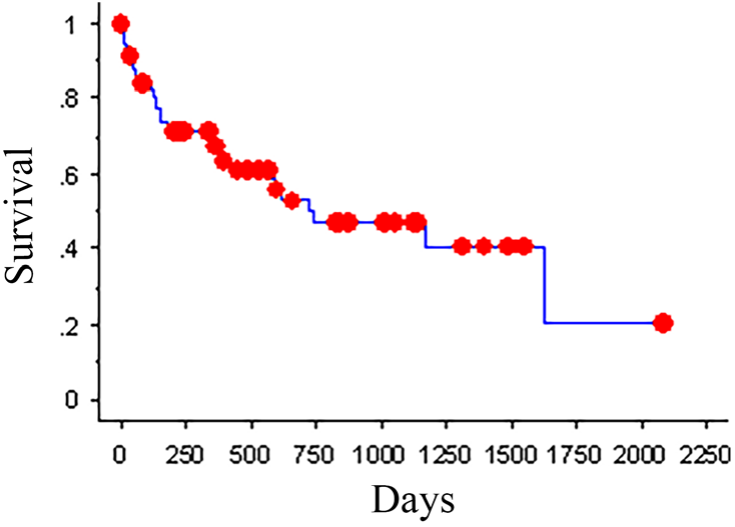
Kaplan‐Meier survival plot for dogs with intracranial neoplasia treated with CyberKnife radiation therapy. Kaplan‐Meier overall median survival time was 738 days. The dots represent censored dogs

**FIGURE 4:**
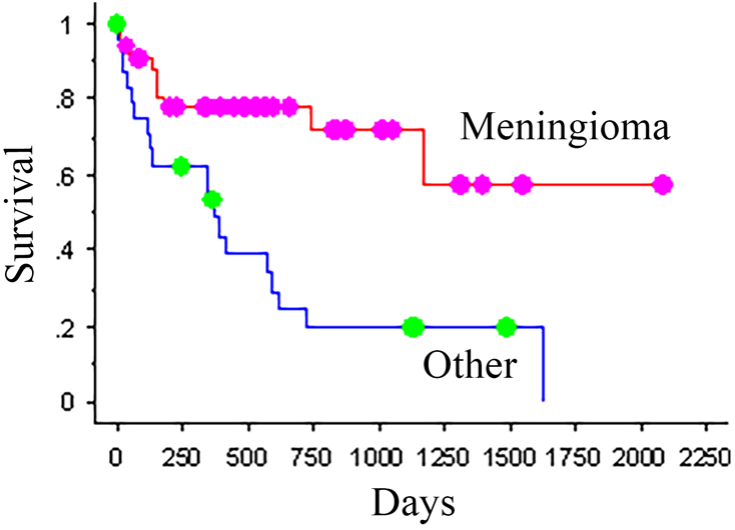
Kaplan‐Meier survival plot for dogs with meningiomas and those with other tumor types treated with CyberKnife radiation therapy. The dots represent censored dogs

The cause of death or reason for euthanasia was tumor related in 28 dogs. At the time of study closure, 7 dogs were still living, and 8 dogs were excluded due to loss of follow‐up. The cause of death or reason for euthanasia was unknown for the remaining 16 dogs. No necropsies were performed. The MST was 738 days (KM analysis, Figure 3). The ST of dogs with presumed meningiomas vs dogs with all other tumor types was compared. Thirty‐three dogs were diagnosed with presumed meningiomas based on advanced imaging. Of these dogs, 6 were still alive at the time of study closure, and 5 were censored due to loss of follow‐up. The MST for the 22 remaining dogs with meningiomas was >2079 days, with the median not yet reached. This was significantly greater than the MST of dogs diagnosed with other tumor types, which was 369 days (KM analysis, *P* < .001; Cox PH univariate analysis, a hazard of 3.675, 95% confidence interval, Figure 4). Of these dogs, 1 was still alive at the time of study closure, and 3 were censored due to loss of follow‐up. Tumor location also significantly impacted MST when tumors were compared based on intra‐axial or extra‐axial location. Dogs with extra‐axial tumors had significantly longer MSTs, >2079 days with the median not yet reached, than those with intra‐axial tumors, who had an MST of 369 days (KM analysis, *P* = .002; Cox PH univariate analysis, a hazard of 3.135, 95% confidence interval). It should be noted that neither tumor type nor tumor location retained significance on Cox PH survival multivariate stepwise analysis.

### 3.4 Adverse events

No adverse events were observed during treatment. One dog, treated for a pituitary macroadenoma, had visual deficits 21 months after radiation. These deficits were thought to be secondary to optic chiasm radiation. No additional adverse events were reported.

## 4 DISCUSSION

In our study, the PFI for dogs with intracranial tumors treated with CyberKnife radiation therapy was 347 days, and the MST was 738 days. This is a similar to possibly improved MST as compared to other published MSTs for dogs with intracranial tumors treated with SRT.^4^, ^7^, ^8^, ^13^ Discernible differences in outcome between different types of SRT have not been identified. Further studies, ideally head‐to‐head, are needed to allow for comparison. The benefits of CyberKnife are largely related to the ability to maximize accuracy in tumor targeting and concurrently minimize damage to normal tissues. Frequent onboard imaging (orthogonal radiographs obtained approximately every 30 seconds), 6D skull tracking, continuous correction for movement, and the large number of beams produced make it well suited for treating intracranial tumors. When compared to traditional fractionated radiation, SRT allows for a higher dose per fraction, sparing of normal tissues to a greater degree, and fewer anesthetic events.

In our study, the biologically effective dose (BED) of late‐responding tissues compares favorably with that of traditional fractionation protocols. Assuming an α/β ratio of 3 for late‐responding tissues, the BED was 88 Gy for the majority of dogs treated in our study.^19^ The conventional fractionated protocol used in the authors' practice of 2.5 Gy for 19 fractions results in a BED of 87 Gy. Therefore, the radiation protocol used in the present study supports the notion that improved tumor response may be observed without consequent increase risk of late‐responding tissue toxicity. Thus, our findings add value to previously published studies and support the use of SRT when available.

When starting our study, we hypothesized that meningiomas treated with CyberKnife radiation therapy would have a greater MST when compared to other types of intracranial tumors treated with SRT. Previous findings indicate that extra‐axial tumors, of which meningiomas are the most common, may respond better to radiation than intra‐axial tumors.^20^, ^21^ We expected that this same trend would be followed for dogs treated with CyberKnife. The MST for meningiomas alone was >2079 days, while the MST for all other tumor types in our study was 369 days. It should be noted that at the close of our research, 7 dogs were still living, and 6 of these were diagnosed with a meningioma. Furthermore, these findings might be impacted by the fact that our diagnoses were based on imaging alone. There are data that show the imaging characteristics of meningiomas are very similar to intracranial histiocytic sarcomas.^22^ While intracranial histiocytic sarcomas are uncommon, the prognosis associated with them is quite poor.^23^ Finally, it could be suggested that the imaging‐based diagnoses in our study make any comparison based on tumor type problematic.

A comparison of intra‐axial vs extra‐axial tumor location revealed a significant improvement in MST for dogs with extra‐axial tumors. Dogs with extra‐axial tumors had MSTs of >2079 days, while dogs with intra‐axial tumors had MSTs of 369 days. This is likely associated with tumor type, as meningiomas are extra‐axial and expected to result in a longer MST. In our study, 32 of the 42 extra‐axial tumors were suspect meningiomas. Of the remaining tumors, 8 were suspect pituitary macroadenomas, 2 were suspect choroid plexus tumors, and 1 was suspected to be either a basilar meningioma or a pituitary macroadenoma. Evaluating the response of pituitary tumors alone could be of some value as functional pituitary tumors have been shown to have a less favorable response to SRT.^24^ Further assessment of the impact of tumor location was made by comparing tumors of the cerebrum, cerebellum, and brainstem. Cerebellar tumors were associated with a significantly shorter PFI than other tumors (97 days for cerebellar tumors vs 266 days for brainstem tumors and 357 days for cerebral tumors).

We examined other prognostic factors, looking for an association that resulted in increased survival. When breed was assessed, golden retrievers were the most common and more likely to be diagnosed with a meningioma. This finding is consistent with previous research which has shown that golden retrievers have a higher incidence of primary intracranial neoplasia and dogs weighing 15 kg or more are more likely to be diagnosed with a meningioma.^25^

The median PFI in our study was 347 days. Because of the variable nature of follow‐up for dogs in this study, it is difficult to know if all recurrent clinical signs were reported. Additionally, only 22 dogs had follow‐up imaging and the timing of this imaging was not standardized. Imaging was performed due to recurrence of clinical signs in 13 cases. When clinical signs recurred, and imaging was not performed, we presumed the intracranial disease was progressive. However, without follow‐up imaging we cannot exclude the possibility that these clinical signs were secondary to late radiation effects. Moving forward, standardization of follow‐up and further investigation into management and treatment of recurrent clinical signs is warranted. Perhaps, long‐term medical management or even prophylactic medical management is appropriate in these cases to extend PFIs.

Only 1 adverse event was reported in our study. Although CyberKnife protocols have been shown to have an improved adverse effect profile when compared to fractionated radiation protocols, our findings are lower than anticipated.^10^ These findings may reflect lack of follow‐up data rather than a truly lower risk of adverse effects.

Our study has several limitations because of its retrospective nature. These include variation in medical management (before and after CyberKnife treatment), nonstandardized follow‐up, lack of definitive diagnoses, and inability to make comparisons between dogs treated with CyberKnife vs other SRT options.

## CONFLICT OF INTEREST DECLARATION

Authors declare no conflict of interest.

## OFF‐LABEL ANTIMICROBIAL DECLARATION

Authors declare no off‐label use of antimicrobials.

## INSTITUTIONAL ANIMAL CARE AND USE COMMITTEE (IACUC) OR OTHER APPROVAL DECLARATION

Authors declare no IACUC or other approval was needed.

## HUMAN ETHICS APPROVAL DECLARATION

Authors declare human ethics approval was not needed for this study.
